# Shock in Adult-Onset Still's Disease Complicated by Macrophage Activation Syndrome: Clinical Characteristics and Prognosis of 14 Patients

**DOI:** 10.1155/ijh/3527708

**Published:** 2025-11-10

**Authors:** Dongxu Li, Lin Cheng, Yaqin Zhang, Long Qian

**Affiliations:** Department of Rheumatology and Immunology, the Second Affiliated Hospital of Anhui Medical University, Hefei City, Anhui Province, China

**Keywords:** adult-onset Still's disease, hypotension, macrophage activation syndrome, shock

## Abstract

**Background:**

Shock is a rare life-threatening complication in patients with adult-onset Still's disease (AOSD) complicated by macrophage activation syndrome (MAS). This study aims to summarize the clinical characteristics and prognosis of this condition.

**Methods:**

We performed a retrospective study of 14 patients hospitalized from January 2017 to December 2023 who experienced shock secondary to AOSD complicated by MAS.

**Results:**

Among the 14 patients, 11 presented with a progressive rash, and 10 exhibited a remittent fever prior to shock onset. Compared to levels at admission, serum ferritin (SF), lactate dehydrogenase (LDH), and procalcitonin levels were significantly elevated, while serum albumin (ALB) levels were significantly decreased, both at the time of MAS diagnosis and at the onset of shock. Shock resolved in nine patients, and five died. All the improved patients were treated with high-dose glucocorticoids (equivalent prednisolone dose ≥ 1 mg/kg/day) prior to MAS diagnosis, and received glucocorticoid pulse therapy. Among them, five (55.6%) received combination therapy with low-dose etoposide and cyclosporine, one (11.1%) received etoposide alone, one (11.1%) received a combination of cyclosporine and tocilizumab, and two (22.2%) receive no additional immunosuppressive therapy. Of the five patients who died, four (80%) received glucocorticoids therapy (two before MAS diagnosis), and only two received a combination of cyclosporine. None of the deceased patients received tocilizumab or etoposide. Compared to patients who died, the patients who improved had significantly higher rates of early glucocorticoid therapy (100% vs. 40%, *p* = 0.027) and etoposide use (66.7% vs. 0%, *p* = 0.028).

**Conclusion:**

Shock is a life-threatening condition secondary to AOSD with MAS. The early combined use of high-dose glucocorticoids and etoposide may be key to improving outcomes in this critical condition.

## 1. Introduction

Adult-onset Still's disease (AOSD) is a rare autoinflammatory disorder primarily involving hyperactivated macrophages and T-cells, along with their associated inflammatory cytokines [1]. The classic clinical manifestations of AOSD include recurrent fever, skin rashes, arthritis, sore throat, splenomegaly, and lymphadenopathy [2].

Macrophage activation syndrome (MAS) is a rare but life-threatening complication which occurs in 15% of patients with AOSD [3]. Shock is a further rare and critical complication in patients with AOSD complicated by MAS. However, reports on AOSD patients who develop shock remain exceedingly scarce [4–9]. This lack of data poses a significant challenge for clinicians in both identifying and treating this severe condition. Therefore, this study aims to summarize the clinical characteristics and outcomes of these patients to provide new insights for the diagnosis and treatment of this condition.

## 2. Methods

### 2.1. Patients and Data Collection

This retrospective study reviewed the clinical data of 14 patients with AOSD complicated by MAS who developed shock during hospitalization in the rheumatology and immunology department or intensive care unit (ICU). All patients were admitted to the Second Affiliated Hospital of Anhui Medical University between January 2017 and December 2023.

All patients met the Yamaguchi criteria for AOSD [10], and malignancies, infections, and other rheumatic diseases were excluded. MAS was diagnosed according to the 2016 European League Against Rheumatism/American College of Rheumatology/Paediatric Rheumatology International Trials Organisation (EULAR/ACR/PRINTO) classification criteria for Systemic Juvenile Idiopathic Arthritis (SJIA) with MAS [11]. Diagnosis required a serum ferritin (SF) level > 684 ng/mL and any two of the following criteria: platelet (PLT) count ≤ 181 × 10^9^/L, aspartate aminotransferase (AST) > 48 U/L, triglyceride (TG) > 156 mg/dL, or fibrinogen (FIB) ≤ 360 mg/dL.

The diagnosis of shock was based on the 2014 European Society of Intensive Care Medicine consensus on circulatory shock and hemodynamic monitoring [12], defined by the presence of arterial hypotension (systolic blood pressure of < 90 mmHg, or mean arterial pressure < 65 mmHg, or a decrease of ≥ 40 mmHg) accompanied by signs of hypoperfusion. These signs included (1) skin that is cold, clammy and blue, pale or discolored; (2) decreased urine output, < 0.5 mL/kg/h; (3) altered mental status characterized by obtundation, disorientation, and confusion. Lactate levels are > 2 mEq/L (or mmol/L). Cardiogenic shock was ruled out by normal echocardiography after shock, and septic shock was excluded through negative infectious etiology workup after shock.

Data collected included demographic and clinical characteristics—age, sex, disease course, time from disease onset to MAS diagnosis, time from MAS diagnosis to shock onset, and the presence of classic AOSD features (fever, sore throat, arthralgia, rash, lymphadenopathy, splenomegaly, and hepatomegaly). Laboratory data was collected at three time points: admission, MAS diagnosis, and shock onset. These parameters included a completed blood count, liver and renal function tests, serum albumin (ALB), lactate dehydrogenase (LDH), TG, FIB, erythrocyte sedimentation rate (ESR), C-reactive protein (CRP), SF, interleukin-6 (IL-6), procalcitonin (PCT), and results of bone marrow examination for hemophagocytosis. Treatment details recorded covered glucocorticoid (GC) use (time to initiation, maximum dose), immunosuppressants, vasoactive agents, and final patient outcomes.

### 2.2. Statistical Analysis

A comparative analysis was performed of laboratory parameters at admission, MAS diagnosis, and shock onset within the same patient cohort. Additionally, clinical characteristics and treatment details were compared between patients who improved and those who died.

Statistical analysis was performed using IBM SPSS 22.0 software. Categorical variables were presented as frequencies with percentages (*n*, %), and the chi-square test was used for the analysis. Moreover, continuous variables were presented as means (standard deviations) or medians (interquartile ranges [IQR]), and Student's *t* test and Mann–Whitney *U* test were used for comparison. Statistical significance was set at *p* < 0.05.

## 3. Results

### 3.1. Clinical Characteristics of Patients

A total of 14 patients were included in this study. All patients were in their first episode and were newly diagnosed. The demographic and clinical characteristics of patients are summarized in Table 1. All patients presented with fever, rash, and lymphadenopathy. The mean time from disease onset to MAS diagnosis was 23.4 ± 12.2 days. Shock occurred within 2 days following MAS diagnosis in 12 patients (85.7%), and the remaining two patients were diagnosed with MAS 2 days after the onset of shock.

**Figure 1:**
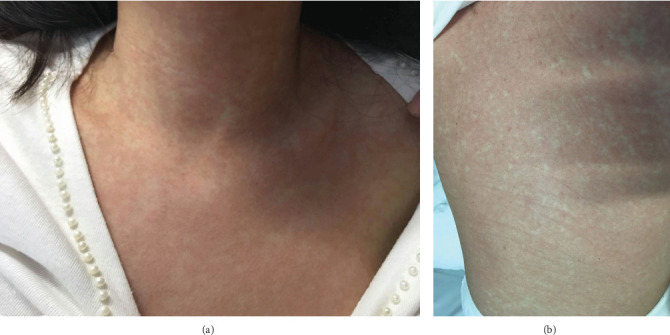
(a) Aggravating, diffuse, deep red, congestive rash on front chest before shock. (b) Aggravating, diffuse, deep red, congestive rash on the trunk before shock.

Notably, 11 patients (78.6%) exhibited a progressive rash before the onset of shock which presented as a diffuse, deep red, congestive rash on the trunk (Figure 1A,B). A remittent fever was observed in 10 patients (71.4%) 1–2 days prior to shock, during which body temperatures failed to normalize.

### 3.2. Laboratory Characteristics of Patients

The laboratory findings at admission, MAS diagnosis, and shock onset are summarized in Table 2 (presented as means or medians).

Compared to levels at admission, the following parameters were significantly lower at the time of MAS diagnosis: white blood cell (WBC) count, absolute neutrophil count (ANC), PLT, ALB, IL-6, and FIB. In contrast, AST, LDH, TG, SF, and PCT levels were significantly elevated.

At shock onset, PLT, ALB, CRP, and FIB levels were significantly lower than admission values, while alanine aminotransferase (ALT), AST, LDH, TG, SF, and PCT levels were significantly higher.

A comparison between shock onset and the time of MAS diagnosis revealed significantly higher WBC and absolute lymphocyte count (ALC) at shock onset, alongside significantly lower ALB levels. No other laboratory parameters showed significant differences between these two time points.

### 3.3. Treatment and Outcome of Patients

The treatments administered and patient outcomes are detailed in Table 3. All patients were treatment-naive. Of the 14 patients, shock resolved in nine, and the other five died of irreversible shock and multiple organ failure despite active rescue efforts in the intensive care unit (ICU).

All 14 patients received antibiotic treatment. All nine patients who improved had been treated with high-dose glucocorticoids (equivalent prednisolone dose ≥ 1 mg/kg/day) prior to MAS diagnosis and shock onset, and subsequently received glucocorticoid pulse therapy (dexamethasone or methylprednisolone, equivalent maximum corticosteroid dose > 120 mg/day prednisolone). Among these nine who improved, five (55.6%) received a combination of low-dose etoposide (one or two 0.1 g doses) and cyclosporine, one (11.1%) received etoposide (in combination with glucocorticoids), one (11.1%) received a combination of cyclosporine and tocilizumab, and two (22.2%) received no additional immunosuppressive agents beyond glucocorticoids.

Of the five patients who died, four (80%) received glucocorticoid therapy (two initiated treatment before MAS diagnosis), and only two received cyclosporine (in combination with glucocorticoids). None received tocilizumab or etoposide.

Seven patients received vasopressors as anti-shock therapy. The vasopressor regimens included: norepinephrine monotherapy (*n* = 2), dopamine monotherapy (*n* = 1), a combination of epinephrine, norepinephrine, and dopamine (*n* = 2), a combination of epinephrine, norepinephrine, dopamine, and metaraminol (*n* = 1), and a combination of epinephrine, norepinephrine, dopamine, dobutamine, and metaraminol (*n* = 1). The maximum recorded infusion rates for these agents were as follows: epinephrine 1.6 *μ*g/kg/min, norepinephrine 3.28 *μ*g/kg/min, dopamine 55.56 *μ*g/kg/min, dobutamine 19.05 *μ*g/kg/min, and metaraminol 3.57 *μ*g/kg/min. Shock resolved in four of these seven patients; three died.

### 3.4. Comparison of Characteristics and Treatments Between Patients Who Improved and those Who Died

A comparison of clinical features, laboratory parameters at shock onset, and treatments between patients who improved and those who died is presented in Table 4.

No significant differences were observed in clinical features between the two groups. At the onset of shock, patients who died exhibited higher median levels of SF (44,417.8 ± 39,827.3 ng/mL) and LDH (1411.0 [970.5–2623.5] U/L) compared to those who improved (SF: 25,273.4 ± 24,617.8 ng/mL; LDH: 857.0 [684.5–1736.0] U/L). However, these differences did not reach statistical significance (*p* > 0.05).

Regarding treatment, a significantly higher proportion of improved patients received early GC therapy (initiated prior to both MAS diagnosis and shock onset) compared to deceased patients (100% vs. 40%, *p* = 0.027). Although the maximum GC dose was higher in improved group, the difference was not statistically significant. Furthermore, low-dose etoposide therapy was administered significantly more frequently to improved patients than to deceased patients (66.7% vs. 0%, *p* = 0.028). No significant differences were found in the use of cyclosporine or tocilizumab between the groups.

## 4. Discussion

Shock is a rare but life-threatening complication of AOSD. A literature search on PubMed yielded only a few studies specifically addressing shock in this patient population [4, 6–9]. In this study, we summarized, for the first time, the clinical features, laboratory characteristics, treatment strategies, and outcomes of shock in patients presenting with AOSD associated with MAS. To the best of our knowledge, this is the largest retrospective study focusing on this critical subset of patients. Our findings aim to provide clinicians with valuable insights to facilitate the diagnosis and management of this condition.

Néel et al. analyzed 20 patients with AOSD admitted to the ICU for AOSD-related organ failure, of whom 10 developed shock. In that cohort, the shock was categorized as non-cardiogenic shock in five, cardiogenic shock in four, and mixed in one [4]. In contrast, the etiology of shock in our patients, while unclear, showed no evidence of a cardiogenic, obstructive, or septic origin. This finding is consistent with case reports by Mejjad et al. and Masui-Ito et al., who also found no evidence of cardiogenic or septic shock in their patients with AOSD [5, 7]. They hypothesized that severe systemic inflammatory response syndrome (SIRS) secondary to AOSD may lead to increased vascular permeability, serving as a direct cause of shock.

In our study, all 14 patients with AOSD had new-onset AOSD and presented with typical manifestations, including spiking fever (100%), rash (100%), sore throat (78.6%), and arthritis (71.4%). However, splenomegaly appeared less common, occurring in only five patients (35.7%). This is noteworthy because splenomegaly has been previously reported as a salient feature of MAS and a potential indicator of poor prognosis in AOSD [13, 14]. For instance, Bae et al. reported a significantly higher frequency of splenomegaly in AOSD patients with reactive hemophagocytic syndrome (RHS) than in those without [14]. Contrary to these previous findings, all five patients with splenomegaly in our cohort improved, and none of the patients who died had developed splenomegaly. This discrepancy suggests that the prognostic significance of splenomegaly may differ in the specific context of AOSD-MAS complicated by shock.

The classic fever pattern in patients with AOSD is high spiking quotidian fever, typically characterized by two daily peaks that resolve within hours. The characteristic rash is an evanescent, salmon-colored, macular, and non-pruritic lesion [15]. Other atypical rash morphologies reported including dermographism, lichenoid, dermatomyositis-like lesions, flagellate, and persistent pruritic eruptions [16–20]. Notably, dermatomyositis-like and persistent pruritic rashes have been associated with a poorer prognosis in AOSD. Skin mottling may be a manifestation of septic shock, which manifests as a diffuse, blanching, reticular, macular, and erythematous lacy rash [21, 22].

In our cohort, most patients developed a persistent high fever (10/14) and a progressive rash (11/14) 1 to 2 days prior to the onset of shock. Notably, their body temperatures failed to normalize despite GC treatment. The rash presented as a diffuse, deep red, congestive eruption on the trunk and limbs (Figure 1A,B), which was distinct from all previously described morphologies. The precise etiology and mechanism underlying these prodromal signs remain elusive. Nevertheless, the emergence of a persistent high fever refractory to GCs and a progressive, atypical rash should alert clinicians to the impending risk of shock.

Our data demonstrated a significant rise in PCT and a concurrent decline in ALB after the development of MAS, a trend that persisted at shock onset. This finding is consistent with prior case reports. Masui-Ito et al. and Agarwal et al. both reported acute hypoalbuminemia in AOSD patients with shock or multi-organ failure [7, 8]. Hypoalbuminemia has been identified as an independent risk factor for increased mortality in cardiogenic shock [23]. While PCT is a sensitive biomarker for bacterial sepsis [24], elevated levels are noted across various shock states, including hypovolemic, anaphylactic, cardiogenic, and toxic shock syndrome [25]. Wang et al. reported that the mean PCT level in patients with HLH was 4.02 ± 8.51 ng/mL. This level was higher in patients with acute kidney injury with HLH [26]. Thus, a rising PCT coupled with a falling ALB may serve as a critical indicator of disease progression towards MAS and shock in AOSD, warranting heightened clinical vigilance. Unfortunately, the small sample size precluded a more robust statistical analysis of these parameters.

Ferritin, an acute-phase reactant highly expressed by activated macrophages, serves as a theoretical marker of macrophage activation [27, 28]. While an SF level exceeding five times the upper limit of normal (> 1000 ng/mL) supports an AOSD diagnosis [29–31], levels typically surpass 5000 ng/mL in MAS [32]. In our study, the mean SF level at admission (8257.9 ± 8384.0 ng/mL) was notably high. Moreover, levels rose dramatically at the time of MAS diagnosis and shock onset (38,893.7 ± 39,346.4 ng/mL and 32,110.7 ± 30,848.5 ng/mL, respectively), suggesting an extreme degree of macrophage activation in patients progressing to shock. Although deceased patients had a higher mean SF level than improved patients, the difference was not statistically significant. This suggests that while the absolute SF level is a marker of disease severity, it may not be a reliable independent predictor of mortality in this specific critical setting.

Our results also demonstrated significant hematological and biochemical shifts at MAS diagnosis compared to admission, characterized by decreases in WBC count, absolute ANC, PLT count, and TG, alongside a significant increase in LDH. These changes are consistent with the established hematophagocytic pathology and cellular damage inherent to MAS progression. Notably, these parameters did not worsen further at shock onset; conversely, WBC and absolute lymphocyte count (ALC) levels increased significantly, likely reflecting a positive response to immunomodulatory treatment.

The management of shock in our cohort required extraordinarily high doses of vasopressors, far exceeding the maximum recommendations for refractory shock in international guidelines (e.g., noradrenaline: 0.5–0.75 *μ*g/kg/min; adrenaline: 0.14–0.5 *μ*g/kg/min) [33, 34]. This requirement aligns with other case reports of AOSD-related shock, where similarly high doses of norepinephrine were administered [5–7]. The extreme vasopressor resistance observed strongly suggests that the underlying pathophysiology of shock in AOSD-MAS—potentially severe systemic inflammation leading to profound capillary leak and vasodilation—is distinct and more severe than that of other common shock types.

Glucocorticoids (GCs) form the cornerstone of therapy for both AOSD and MAS. High-dose regimens are standard, with pulse therapy (e.g., methylprednisolone 30 mg/kg/day) recommended for severe MAS, emphasizing that early intervention is crucial for favorable outcomes [35–37]. Our findings strongly support this principle: all improved patients received high-dose GC therapy before MAS diagnosis, a rate significantly higher than in deceased patients. This suggests that early, aggressive GC intervention, prior to the full progression of MAS, may be critical for reversing shock. However, as evidenced by Néel et al. where only 50% of shock patients responded to high-dose GCs, steroid therapy alone is not always sufficient, particularly in advanced stages [4].

For GC-refractory MAS, international guidelines recommend escalating therapy with cyclosporine A (CsA) or agents from the HLH-94/2004 protocol, such as etoposide [38–40]. While over half of our patients received CsA, its use did not significantly differ between outcomes. In stark contrast, the administration of low-dose etoposide was a pivotal differentiating factor: it was used in 66.7% of improved patients and none of the deceased patients. This finding corroborates the work of Wang et al., which demonstrated that short-course, low-dose etoposide can significantly improve survival in refractory AOSD-MAS [41]. Although biologics like anakinra are effective alternatives [42], their limited availability in China makes etoposide a critically important option. Our results posit that early high-dose GCs combined with etoposide may constitute a key therapeutic strategy for shock complicating AOSD-MAS.

### 4.1. Limitations of the Study

This study has several limitations. First, its retrospective design and small sample size, inherent to the rarity of the condition, limit the generalizability of the findings. Consequently, we could not identify independent risk factors for shock in patients with AOSD. Future prospective, multi-center studies with larger cohorts are required to validate our observations and further elucidate the clinical and laboratory characteristics of this critical complication.

## 5. Conclusion

Shock is a rare life-threatening complication secondary to AOSD associated with MAS. A persistent high fever refractory to glucocorticoids and a progressive, atypical rash may serve as critical prodromal signs of impending shock. Laboratory findings such as markedly elevated procalcitonin and acute hypoalbuminemia may signify a deteriorating clinical course. Notably, early intervention with high-dose glucocorticoids, prior to the full establishment of MAS, combined with etoposide therapy, may be the key to improving outcomes in this critical condition.

## Figures and Tables

**Table 1:** Clinical features of 14 patients.

**Clinical features**	**Total (** **n** = 14**)**
Sex, female, *n* (%)	9.00 (64.30)
Age, years, mean ± SD	34.90 ± 14.20
Disease duration to admission, days, mean ± SD	14.10 ± 7.80
Disease duration to MAS, days, mean ± SD	23.40 ± 12.20
Fever^a^, *n* (%)	14.0 (100.00)
Sore throat^a^, *n* (%)	11.00 (78.60)
Arthritis^a^, *n* (%)	10.00 (71.40)
Rash^a^, *n* (%)	14.00 (100.00)
Lymphadenectasis^a^, *n* (%)	14.00 (100.00)
Splenomegaly^a^, *n* (%)	5.00 (35.70)
Remittent fever^b^, *n* (%)	10.00 (71.40)
Progressive rash^b^, *n* (%)	11.00 (78.60)
Bone marrow hemophagocytosis, *n* (%)	6.00 (50.00)

^a^These clinical characteristics were recorded at the time of admission.

^b^These clinical characteristics were recorded at the time of shock onset.

**Table 2:** Laboratory characteristics of 14 patients.

**Laboratory characteristics**	**At admission**	**At MAS**	**At shock**	***p* Value** ^**a**^	***p* Value** ^**b**^	***p* Value** ^**c**^
WBC, × 10^9^/L	17.39 ± 7.13	13.78 ± 5.61	17.57 ± 8.18	0.021∗	0.919	0.046∗
ANC, × 10^9^/L	15.38 ± 7.85	11.94 ± 5.02	12.89 ± 5.38	0.025∗	0.648	0.277
ALC, × 10^9^/L	1.26 ± 0.96	0.65 (0.34, 1.24)	1.60 ± 1.26	0.314	0.380	0.022∗
Hb, g/L	121.08 ± 16.58	118.86 ± 15.04	120.36 ± 15.14	0.717	0.912	0.691
PLT, × 10^9^/L	253.77 ± 79.98	140.44 ± 72.51	161.50 ± 92.38	0.030∗	0.016∗	0.311
ALT, U/L	46.00 (26.25, 99.50)	99.00 (46.00, 205.00)	150.50 (74.25, 197.50)	0.114	0.041∗	0.600
AST, U/L	45.50 (30.50, 130.50)	162.50 (105.75, 201.00)	169.00 (105.75, 226.00)	0.037∗	0.023∗	0.674
LDH, U/L	401.00 (297.50, 737.50)	1248.14 ± 780.48	1266.00 (787.25, 1721.00)	0.008∗∗	0.003∗∗	0.249
TG, mg/dL	108.30 ± 45.47	223.27 ± 144.26	207.53 ± 151.62	0.021∗	0.046∗	0.368
ALB, g/dL	3.09 ± 0.57	2.76 ± 0.65	2.41 ± 0.60	0.030∗	0.001∗∗	0.046∗
Scr, *μ*mol/L	0.83 ± 0.30	1.06 ± 0.59	1.09 ± 0.66	0.123	0.125	0.784
ESR, mm/H	37.23 ± 22.07	20.18 ± 17.86	23.36 ± 18.01	0.097	0.084	0.387
CRP, mg/dL	13.86 ± 9.25	4.63 (2.71, 13.04)	7.18 ± 5.41	0.074	0.028∗	0.575
SF, ng/mL	8257.86 ± 8384.05	38,893.71 ± 39,346.45	32,110.71 ± 30,848.51	0.012∗	0.008∗∗	0.497
IL-6, pg/mL	111.00 (58.55, 301.05)	58.10 (23.84, 98.40)	56.05 (11.75, 221.33)	0.021∗	0.285	0.866
PCT, ng/mL	0.46 (0.11, 1.29)	1.40 (0.51, 5.87)	2.21 (0.51, 5.90)	0.008∗∗	0.008∗∗	0.249
FIB, mg/dL	532.50 ± 168.64	264.71 ± 143.79	241.43 ± 129.63	0.002∗∗	< 0.001∗∗∗	0.350

Abbreviations: ALB, albumin; ALC, absolute lymphocyte count; ALT, alanine aminotransferase; ANC, absolute neutrophil count; AST, aspartate aminotransferase; CRP, C-reactive protein; ESR, estimated sedimentation rate; FIB, fibrinogen; Hb, hemoglobin; IL-6, interleukin-6; LDH, lactate dehydrogenase; MAS, macrophage activation syndrome; PCT, procalcitonin; PLT, platelet; Scr, serum creatinine; SF, serum ferritin; TG, triglyceride; WBC, white blood cell.

∗*p* value < 0.05; ∗∗*p* value < 0.01; ∗∗∗*p* value < 0.001.

^a^ *p* Value compared between time of admission and MAS.

^b^ *p* Value compared between time of admission and shock.

^c^ *p* Value compared between time of MAS and shock.

**Table 3:** Treatment and outcome of 14 patients.

**No.**	**Time from admission to treatment (day)**	**GC/started before MAS**	**CsA**	**TCZ**	**Etoposide**	**Vasopressors and maximum dose (*μ*g/kg min)**	**Outcome**
1	15	Pred 50 mg/no	No	No	No	NE (unknown dosage)	Died
						DA (unknown dosage)	
2	5	MP160 mg/no	No	No	No	AD (0.22) NE (1.11) DA (55.56) Metaraminol(0.83)	Died
3	5	MP500 mg/yes	Yes	No	No	AD (1.60) NE (2.13) DA (26.67)	Died
4	—	No/no	No	No	No	Unknown	Died
5	10	DXM20 mg/yes	Yes	No	No	AD (0.10) NE (2.67) Metaraminol (3.57) Dob (19.05)	Died
6	7	DXM40 mg/yes	Yes	No	Yes	NE (0.21)	Improved
7	1	MP320 mg/yes	Yes	No	Yes	NE (3.28)	Improved
8	1	DXM40 mg/yes	Yes	No	Yes	AD (0.06) NE (2.07) DA (6.47)	Improved
9	6	DXM20 mg/yes	No	No	Yes	DA (3.64)	Improved
10	6	DXM40 mg/yes	Yes	No	Yes	No	Improved
11	1	DXM20 mg/yes	No	No	No	No	Improved
12	3	DXM20 mg/yes	Yes	No	Yes	No	Improved
13	5	DXM20 mg/yes	No	No	No	No	Improved
14	4	MP500 mg/yes	Yes	Yes	No	No	Improved

Abbreviations: AD, adrenaline; CsA, cyclosporine A; DA, dopamine; Dob, dobutamine; DXM, dexamethasone; F, female; GC, glucocorticoid; M, male; MAS, macrophage activation syndrome; MP, methylprednisolone; NE, norepinephrine; Pred, prednisolone; TCZ, tocilizumab.

**Table 4:** Comparison of characteristics between improved patients and those who died.

**Variables**	**Improved patients (** **n** = 9**)**	**Patients who died (** **n** = 5**)**	***p* Value**
Sex, female, *n* (%)	6.00 (66.67%)	3.00 (60.00%)	0.622
Age, years, mean ± SD	33.56 ± 10.57	37.40 ± 20.43	0.710
Sore throat, *n* (%)	7.00 (77.78%)	4.00 (80.00%)	0.725
Arthritis, *n* (%)	6.00 (66.67%)	4.00 (80.00%)	0.545
Rash, *n* (%)	9.00 (100.00%)	5.00 (100.00%)	1.000
Lymphadenectasis, *n* (%)	9.00 (100.00%)	5.00 (100.00%)	1.000
Splenomegaly, *n* (%)	5.00 (55.56%)	0.00 (0.00%)	0.086
Malignant fever, *n* (%)	6.00 (66.67%)	4.00 (80.00%)	0.545
Progressive rash, *n* (%)	7.00 (77.78%)	4.00 (80.00%)	0.725
WBC, × 10^9^/L	16.57 ± 7.83	19.37 ± 9.39	0.560
ANC, × 10^9^/L	13.07 ± 6.77	12.61 ± 2.62	0.867
Hb, g/L	118.11 ± 16.31	124.40 ± 13.46	0.814
PLT, × 10^9^/L	167.56 ± 95.32	150.60 ± 96.60	0.756
ALT, U/L	176.00 (73.50, 292.50)	81.00 (72.50, 198.50)	0.549
AST, U/L	163.00 (81.50, 232.50)	175.00 (137.00, 493.50)	0.549
LDH, U/L	857.00 (684.50, 1736.00)	1411.00 (970.50, 2623.50)	0.549
Scr, *μ*mol/L	0.91 ± 0.30	1.38 ± 0.98	0.360
CRP, mg/dL	7.91 ± 5.96	5.86 ± 4.56	0.519
SF, ng/mL	25,273.44 ± 24,617.82	44,417.80 ± 39,827.27	0.283
FIB, mg/dL	262.44 ± 136.39	203.60 ± 120.93	0.438
Bone marrow hemophagocytosis	4.00 (50.00%)	2.00 (50.00%)	0.727
Maximum GC dose, mg	262.04 ± 164.74	201.67 ± 248.78	0.592
GC started before MAS diagnosis and shock, *n* (%)	9.00 (100.00%)	2.00 (40.00%)	0.027∗
CsA	6.00 (66.67%)	2.00 (40.00%)	0.343
TCZ	1.00 (11.11%)	0.00 (0.00%)	0.643
Etoposide	6.00 (66.67%)	0.00 (0.00%)	0.028∗
Use of antibiotic	9.00 (100.00%)	5.00 (100.00%)	—

Abbreviations: ALT, alanine aminotransferase; ANC, absolute neutrophil count; AST, aspartate aminotransferase; CRP, C-reactive protein; CsA, cyclosporine A; FIB, fibrinogen; GC, glucocorticoid; Hb, hemoglobin; LDH, lactate dehydrogenase; PLT, platelet; Scr, serum creatinine; SF, serum ferritin; TCZ, tocilizumab; WBC, white blood cell.

∗*p* value < 0.05.

## Data Availability

The datasets used and analyzed in the current study are available from the corresponding authors upon request.
